# Resting-State 40 Hz EEG Activity Before and After Single-Session Non-Flickering 40 Hz Light Stimulation in Cognitively Normal Older Adults: An Uncontrolled Pilot Study

**DOI:** 10.3390/brainsci16090976

**Published:** 2026-09-15

**Authors:** Chia-Hsiung Cheng, Hsinjie Lu

**Affiliations:** 1 Department of Occupational Therapy and Graduate Institute of Behavioral Sciences, Chang Gung University, Taoyuan 333323, Taiwan; hsinjie@cgu.edu.tw; 2 Laboratory of Brain Imaging and Neural Dynamics (BIND Lab), Chang Gung University, Taoyuan 333323, Taiwan; 3 Healthy Aging Research Center, Chang Gung University, Taoyuan 333323, Taiwan; 4 Department of Neurology, Chang Gung Memorial Hospital, Kaohsiung 833401, Taiwan

**Keywords:** Alzheimer’s disease (AD), endogenous gamma oscillations, 40 Hz, electroencephalography (EEG), neural entrainment, non-invasive brain stimulation

## Abstract

**Background**: Forty-hertz sensory stimulation has emerged as a potential approach for modulating neural activity relevant to Alzheimer’s disease. However, electrophysiological changes following non-flickering 40 Hz light stimulation in older adults remain unclear. This study investigated whether a single-session intervention of non-flickering 40 Hz light stimulation would be associated with increased resting-state 40 Hz oscillations in cognitively normal older adults. **Methods**: In this uncontrolled single-arm pilot study, 16 cognitively normal older adults underwent a 60 min session of non-flickering 40 Hz light stimulation. Resting-state EEG was recorded immediately before and after stimulation. Relative power within 38–42 Hz was analyzed across six predefined scalp regions and the whole-brain measure using one-tailed Wilcoxon signed-rank tests with Benjamini–Hochberg false discovery rate (FDR) correction. Exploratory real-time EEG recordings during stimulation were available in 10 participants. **Results**: After FDR correction, resting-state 38–42 Hz relative power was higher post-stimulation in the central (FDR = 0.045, effect size = 0.555) and right temporal (FDR = 0.014, effect size = 0.724) regions. In the absolute-power sensitivity analysis, only the right temporal increase remained significant after FDR correction (FDR = 0.042). Exploratory during-stimulation analysis showed a nominally increased 38–42 Hz signal-to-noise ratio in the right temporal region (*p* = 0.026). One participant reported very mild fatigue; no other adverse responses were reported. **Conclusions**: This pilot study suggests regional increases in resting-state 38–42 Hz activity after non-flickering 40 Hz light stimulation, with additional absolute-power support for the right temporal finding. These findings remain preliminary given the uncontrolled design and small sample.

## 1. Introduction

Alzheimer’s disease (AD) is a neurodegenerative disorder characterized by a gradual decline in cognitive function in older adults, eventually impairing their ability to carry out daily activities. The pathological hallmarks of AD include the abnormal accumulation of amyloid-beta (Aβ) and phosphorylated tau proteins, which contribute to neuroinflammation, synaptic dysfunction, and neuronal loss [1,2]. Beyond these classical pathological features, AD is increasingly recognized as a multifactorial neurodegenerative disorder involving widespread intracellular dysfunction. In particular, disruption of mitochondrial homeostasis may contribute to neuronal vulnerability and disease progression. Emerging evidence further suggests that TDP-43, a frequent co-pathology in AD, may exacerbate mitochondrial dysfunction and contribute to the pathological heterogeneity of the disease [3]. Alongside system-level interventions, advances in precision genome engineering, including gene editing and genome-writing strategies, represent emerging molecular-level therapeutic avenues for complex neurodegenerative disorders [4]. Within this broader therapeutic landscape, gamma-frequency sensory stimulation has emerged as a potential non-pharmacological approach for modulating neural activity and AD-related pathology. Previous animal studies have demonstrated that administering 40 Hz (i.e., gamma-frequency) flickering light stimulation to mice for 1 h per day over 7 consecutive days significantly reduced both the amount and area of Aβ deposits in the visual cortex; moreover, 40 Hz light stimulation altered microglial morphology, increasing their diameter [5]. Interestingly, light stimulation at 20 Hz, 80 Hz, or random frequencies did not produce these effects [5]. Subsequent studies further showed that 40 Hz sensory stimulation reduced Aβ burden in the hippocampus and prefrontal cortex and improved visuospatial memory performance [6,7,8]. These promising findings in animal models have motivated researchers to investigate whether similar benefits can be achieved in humans [9,10].

To date, several intervention studies have applied 40 Hz visual or audiovisual stimulation to examine its effects on brain structure, function, Aβ levels, or cognitive outcomes in older adults and individuals with mild cognitive impairments or dementia [11,12,13,14,15,16,17]. For example, Agger and colleagues conducted a randomized, placebo-controlled, double-blind trial with 11 patients with mild to severe dementia, randomly assigned to either an experimental group (40 Hz white light stimulation with invisible spectral flicker) or a control group (continuous non-40 Hz light of matched intensity). Participants received 1 h of stimulation daily for 6 weeks, with follow-up 6 weeks later. Compared to controls, the experimental group showed a near-significant immediate improvement in cognitive function and exhibited larger hippocampal volumes and smaller ventricular volumes at follow-up [11]. Similarly, a team at the Massachusetts Institute of Technology recruited 15 patients with mild probable AD and randomly assigned them to either an experimental group (40 Hz flickering light plus 40 Hz sound stimulation) or a control group (constant light and white noise). The intervention was delivered 1 h daily for 3 months. Results showed that, compared with controls, the 40 Hz stimulation group had reduced hippocampal atrophy, enhanced functional connectivity within the default mode and medial visual networks, better performance on the face–name delayed recall test, and improved daily activity rhythmicity [12]. A more recent study further demonstrated that a 3-month intervention of 40 Hz non-flickering visual stimulation reduced neuropsychiatric symptoms and caregiver burden in patients with AD compared with controls [17]. However, it is notable that none of these human studies used electrophysiological measures to directly assess changes in gamma-band spectral power before and after the intervention.

From a methodological perspective, numerous observational studies have shown that compared with non-flickering light, flickering light can entrain stronger spectral power at the stimulation frequency [18,19]. In intervention trials, some research groups have used 40 Hz flickering light [12,13,15,16], whereas others have developed non-flickering (i.e., invisible) 40 Hz light technology [11,17]. The advantage of the non-flickering approach is improved comfort, as participants are not exposed to visible flicker. Although prior studies using non-flickering 40 Hz stimulation have reported potential improvements in cognition and neuropsychiatric symptoms [11,17], direct electrophysiological evidence demonstrating increased 40 Hz spectral power following such interventions is still lacking.

To establish feasibility and assess the immediate neurophysiological effects of 40 Hz non-flickering visual stimulation, a single-session design was adopted in this pilot study. This approach allows us to minimize participant burden, reduce potential safety concerns, and provide a controlled framework for evaluating whether acute changes in gamma-band activity can be reliably detected. Demonstrating short-term modulation of neural oscillations is a critical first step before progressing to longer and more intensive intervention protocols aimed at cognitive or clinical outcomes. In addition, evidence from animal and human studies suggests that gamma entrainment can occur immediately after stimulation, supporting the rationale for testing the efficacy of a one-session paradigm [20,21].

In summary, this pilot study recruited cognitively normal older adults to participate in a single 60 min session of 40 Hz non-flickering light stimulation. Resting-state 40 Hz spectral power was measured using EEG immediately before and after the intervention.

## 2. Methods

This study was approved by the ethics committee of the Chang Gung Memorial Hospital (No.: 202401421B0A3). All experiments were carried out in accordance with relevant guidelines and regulations (Declaration of Helsinki), and all participants provided written informed consent before participating in this study.

This study was an uncontrolled single-arm pre–post pilot analysis of participants receiving 40 Hz non-flickering light stimulation within an ongoing randomized controlled trial registered at ClinicalTrials.gov (NCT06715995; https://clinicaltrials.gov/study/NCT06715995 (accessed on 10 September 2026); registered on 4 December 2024). The present report focuses on preliminary findings from the intervention group only, examining pre- and post-intervention changes. Full trial results, including comparisons with the control group and long-term outcomes, will be reported separately after study completion.

### 2.1. Participants

We recruited 16 healthy, community-dwelling older adults (12 females; mean age = 70.56 years; mean years of education = 11.43) to participate in this study. Inclusion criteria were: (1) age 55 years or older; (2) a score on the Cognitive Abilities Screening Instrument (CASI) within the normal range after adjustment for age and education; (3) self-reported absence of serious neurological or psychiatric disorders that could affect cognitive function (e.g., stroke, epilepsy, depression, migraine); (4) not currently taking medications known to influence cognitive function (e.g., benzodiazepines, anticholinergics); and (5) normal or corrected-to-normal vision. Exclusion criteria included: (1) participation in other cognition-enhancing studies within the past two months and (2) medical conditions potentially affecting cognitive function (e.g., cancer, autoimmune diseases). Table 1 shows the detailed demographic and neuropsychological information of the included subjects.

The present study represents a preliminary analysis of the first 16 participants in the 40 Hz intervention arm who were recruited between January and June 2025 and had completed the single-session intervention and corresponding pre- and post-intervention EEG assessments at the time of data extraction. All available participants meeting these criteria were included, and participants were not selected according to their EEG responses or neuropsychological characteristics. No formal power calculation was performed specifically for this preliminary single-arm analysis; the sample size was determined by the number of eligible participants with completed pre- and post-intervention EEG assessments available at the time of data extraction and did not represent a predetermined stopping point or prospectively specified interim sample size. Resting-state EEG assessment of 40 Hz activity was prospectively specified as a primary outcome of the registered trial.

### 2.2. Intervention

A custom-designed vertical LED light source (Aleddra, Inc., Seattle, WA, USA), with a panel size of 16 × 22 cm, was used to deliver non-flickering gamma-frequency stimulation. The device comprised two light sources independently driven by square-wave modulation at carrier frequencies of 80 and 120 Hz, respectively, with a 50% duty cycle and 100% modulation depth. Both carrier frequencies were above the critical flicker fusion threshold (>60 Hz), such that the individual high-frequency modulations were not perceived as visible flicker. The optical output itself consisted of the 80- and 120 Hz modulated signals rather than direct 40 Hz modulation. The stimulation paradigm was designed to generate a 40 Hz difference-frequency response (120 − 80 Hz = 40 Hz) through nonlinear processing of the two high-frequency visual inputs within the visual system [22]. To objectively characterize the optical output, the light source was measured using an EVERFINE LFA-3000 Light Flicker Analyzer (EVERFINE Corporation, Hangzhou, China). The optical signal was sampled at 20 kS/s and analyzed in both the time and frequency domains. Time-domain measurements showed high-frequency optical switching without 40 Hz periodicity, whereas frequency-domain analysis showed discrete spectral components at 80 and 120 Hz, with no measurable spectral component at 40 Hz. These measurements confirmed that the experimental light source did not directly emit measurable 40 Hz optical modulation. The light source had a correlated color temperature of 4000 K and was positioned approximately 100 cm from the participant’s eyes, delivering an illuminance of 400 lux at eye level. The visual angle was approximately 45° to either the left or right side.

During the 60 min stimulation session, all participants remained in the laboratory and followed the same general procedure. Participants primarily responded to brief questions from the research staff regarding demographic and related background information, received instructions concerning the subsequent longer-term intervention phase, and engaged in conversation with the research staff. Participants were not assigned to different activity conditions or instructed to engage in self-selected activities during stimulation.

### 2.3. EEG Recordings

Electrophysiological responses were recorded using a dry-electrode, battery-powered, portable DSI-24 EEG headset (WEARABLE Sensing Inc., San Diego, CA, USA). This headset was equipped with 19 scalp electrodes according to the international 10–20 system, including Fp1, Fp2, F3, F4, Fz, F7, F8, C3, Cz, C4, T3, T4, T5, T6, P3, Pz, P4, O1, and O2. In addition, two electrodes (A1 and A2) were placed on the left and right earlobes, respectively, for subsequent offline re-referencing. Eye movements were monitored using two electrooculography (EOG) electrodes positioned above the left orbit and below the right orbit. During EEG acquisition, Pz served as the online reference, and the sampling rate was set at 300 Hz [23]. Electrode–scalp contact was optimized using the DSI-24 hair-parting tool, and electrode impedance was maintained below 1 MΩ before data acquisition, consistent with previously reported procedures for this dry-electrode system [24]. Resting-state EEG activity was recorded for each participant immediately before and after the intervention. During the recordings, participants were instructed to remain relaxed and awake with their eyes closed for 5 min. An eyes-closed resting-state condition was selected to minimize ongoing visual inputs and reduce variability related to visual exploration, eye movements, and gaze, thereby providing a standardized condition for assessing intrinsic neural activity before and after stimulation. The same recording condition was used at both time points.

**Figure 1:**
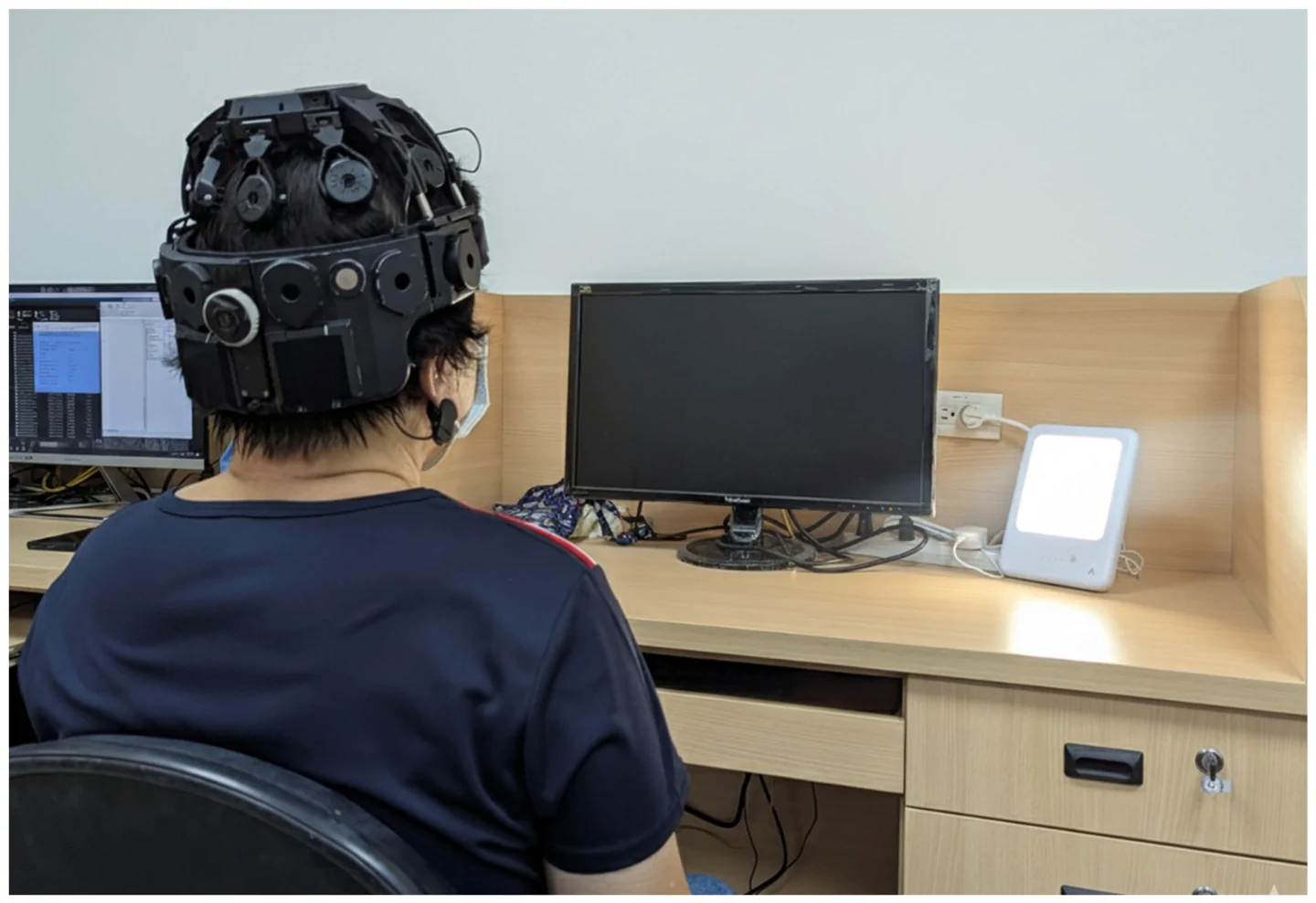
Experimental setup for non-flickering 40 Hz light stimulation and concurrent EEG recording. The participant was seated in front of the light device while EEG was recorded during stimulation. This setup was used for the exploratory assessment of spectral activity during non-flickering 40 Hz light stimulation.

To characterize spectral activity during non-flickering 40 Hz light stimulation, all 16 participants were invited to return for an additional two-minute real-time EEG recording during stimulation (Figure 1). Ten participants were available and willing to return for this additional assessment and therefore comprised the real-time EEG subset. These recordings were analyzed as an exploratory assessment of spectral activity during stimulation and were conducted independently of the primary pre- and post-intervention analyses.

### 2.4. Analysis of Pre- and Post-Stimulation Resting-State 38–42 Hz Power

All preprocessing procedures were performed using EEGLAB [25]. During offline preprocessing, EEG data were re-referenced to the average of the left and right earlobe electrodes (A1 and A2), which served as the final reference for all subsequent EEG analyses. A high-pass filter at 0.1 Hz and a notch filter at 60 Hz were applied. When bad channels were identified, they were reconstructed using EEGLAB’s channel-interpolation function. Each 5 min resting-state EEG recording was segmented into 2 s epochs. EEG epochs were first screened using an amplitude criterion of ±100 μV, followed by visual inspection of all epochs to determine whether they were retained or rejected. Independent component analysis (ICA) was then applied to reduce artifacts. ICA components were visually reviewed by an experienced EEG researcher based on their scalp topography and temporal activity patterns, and components identified as artifactual were removed. The same preprocessing and artifact-rejection procedures were applied to both pre- and post-intervention EEG data. Following artifact rejection, a mean of 132.4 ± 4.3 epochs was retained for the pre-intervention recordings and 130.0 ± 5.2 epochs (*Z* = 0.653, *p* = 0.514) for the post-intervention recordings.

Following preprocessing, power spectral density for each channel was subsequently computed from the retained artifact-free epochs using Analyzer 2.3 (BrainVision, Brain Products GmbH, Gilching, Germany). A Fast Fourier Transform (FFT) was applied using a Hanning window (window length: 50%) and a spectral resolution of 0.5 Hz. Relative spectral power was first calculated in BrainVision Analyzer for each frequency bin by normalizing the absolute power at that frequency to the total spectral power within the 1–100 Hz frequency range. Specifically, for each electrode, relative power at frequency f was calculated as

$$RP\left(f\right)=\frac{P\left(f\right)}{\sum_{f^{\prime }\in \left[1,100\right]}P\left(f^{\prime }\right)}$$

where P(f) represents the absolute spectral power at frequency f, and f′ is the frequency-bin index used to sum the spectral power across all frequency bins within the 1–100 Hz range. Resting-state 38–42 Hz relative power was then quantified by averaging the relative-power values across all frequency bins within the predefined 38–42 Hz range:

$$RP_{38–42}=\frac{1}{N}\sum_{f\in \left[38,42\right]}RP\left(f\right)$$

where N represents the number of frequency bins within the 38–42 Hz range. With the spectral resolution of 0.5 Hz used in the present analysis, the calculation included the frequency bins from 38 to 42 Hz at 0.5 Hz intervals.

For each participant, the resulting 38–42 Hz relative-power values were first calculated for each electrode and subsequently averaged within six predefined scalp regions: frontal (Fp1, Fp2, F3, F4, Fz, F7, F8), central (C3, Cz, C4), parietal (P3, Pz, P4), left temporal (T3, T5), right temporal (T4, T6), and occipital (O1, O2). A whole-brain measure was additionally calculated by averaging the corresponding values across all 19 electrodes. These six regional measures and the whole-brain measure were used for the pre–post statistical comparisons.

Because relative spectral power is dependent on the total-power normalization denominator, an additional sensitivity analysis was performed using absolute 38–42 Hz power to assess whether the observed pre–post differences in relative power could be explained solely by changes in the normalization denominator. Absolute 38–42 Hz power was quantified by averaging the absolute spectral-power values across the same 38–42 Hz frequency bins and using the same electrode and scalp-region definitions as in the relative-power analysis. Pre–post differences in absolute power were evaluated using the same statistical framework as the primary relative-power analysis.

### 2.5. Exploratory Analysis of EEG Activity During Stimulation

For the subset of 10 participants with real-time EEG recordings during stimulation, an exploratory signal-to-noise ratio (SNR) analysis was performed to quantitatively assess spectral prominence around the target stimulation frequency. The signal was defined as the mean spectral power across the predefined 38–42 Hz frequency range, without selecting individual spectral peaks. Noise was estimated as the mean spectral power across the immediately adjacent 1 Hz frequency ranges below and above the target band (37–38 Hz and 42–43 Hz, respectively), excluding frequency bins included in the 38–42 Hz signal range. SNR was calculated as the ratio of the mean 38–42 Hz power to the mean power across the flanking frequency ranges. The same SNR calculation was applied to the pre-stimulation and during-stimulation recordings for each participant. SNR values were calculated for the six predefined scalp regions and the whole-brain measure.

### 2.6. Assessment of Adverse Responses

Adverse events were assessed at the end of the intervention. Participants completed a standardized form that recorded the presence or absence of specific adverse effects, including fatigue, headache, dizziness, dazzling, ocular pain, and other symptoms. For each reported symptom, participants indicated occurrence (“Yes” or “No”) and, if present, rated its severity on a 5-point Likert scale (1 = very mild, 2 = mild, 3 = tolerable, 4 = severe, 5 = very severe).

### 2.7. Statistical Analysis

All statistical analyses were performed using IBM SPSS Statistics, version 22 (IBM Corp., Armonk, NY, USA). Continuous data are presented as mean ± standard error of the mean (SEM), together with median and interquartile range.

Given the small sample size and the non-parametric analytical framework adopted for this preliminary analysis, pre–post differences in resting-state 38–42 Hz relative power were evaluated using Wilcoxon signed-rank tests. Based on the a priori directional hypothesis that resting 38–42 Hz power would be higher following 40 Hz non-flickering light stimulation, one-tailed Wilcoxon signed-rank tests were used to compare post-intervention with pre-intervention values. To account for multiple comparisons, *p* values across all seven EEG measures—the six predefined scalp regions (frontal, central, parietal, left temporal, right temporal, and occipital) and the whole-brain measure—were adjusted using the Benjamini–Hochberg false discovery rate (FDR) procedure. Although the whole-brain measure was derived from the same electrodes comprising the six regional measures and was therefore not statistically independent of them, it was retained within the correction family to provide a conservative adjustment across all reported EEG comparisons. FDR < 0.05 was considered statistically significant.

As a sensitivity analysis, pre–post differences in absolute 38–42 Hz power were evaluated using the same statistical framework as the primary relative-power analysis, including one-tailed Wilcoxon signed-rank tests and Benjamini–Hochberg FDR correction across the same seven EEG measures.

For the exploratory real-time EEG analysis in the subset of 10 participants, SNR values during stimulation were compared with pre-stimulation values using one-tailed Wilcoxon signed-rank tests, based on the a priori directional hypothesis that 38–42 Hz spectral prominence would be greater during 40 Hz stimulation than before stimulation. Because this analysis was exploratory and was not part of the primary outcome analysis, unadjusted *p* values are reported and interpreted accordingly.

For the primary relative-power analysis and absolute-power sensitivity analysis, effect sizes for the Wilcoxon signed-rank tests were calculated as *r* = |*Z*|/√*N*, where *Z* represents the standardized test statistic and *N* represents the number of non-zero paired observations. Hodges–Lehmann (HL) estimates of paired changes with 95% confidence intervals were additionally reported to quantify the magnitude and precision of the observed changes.

## 3. Results

**Figure 2:**
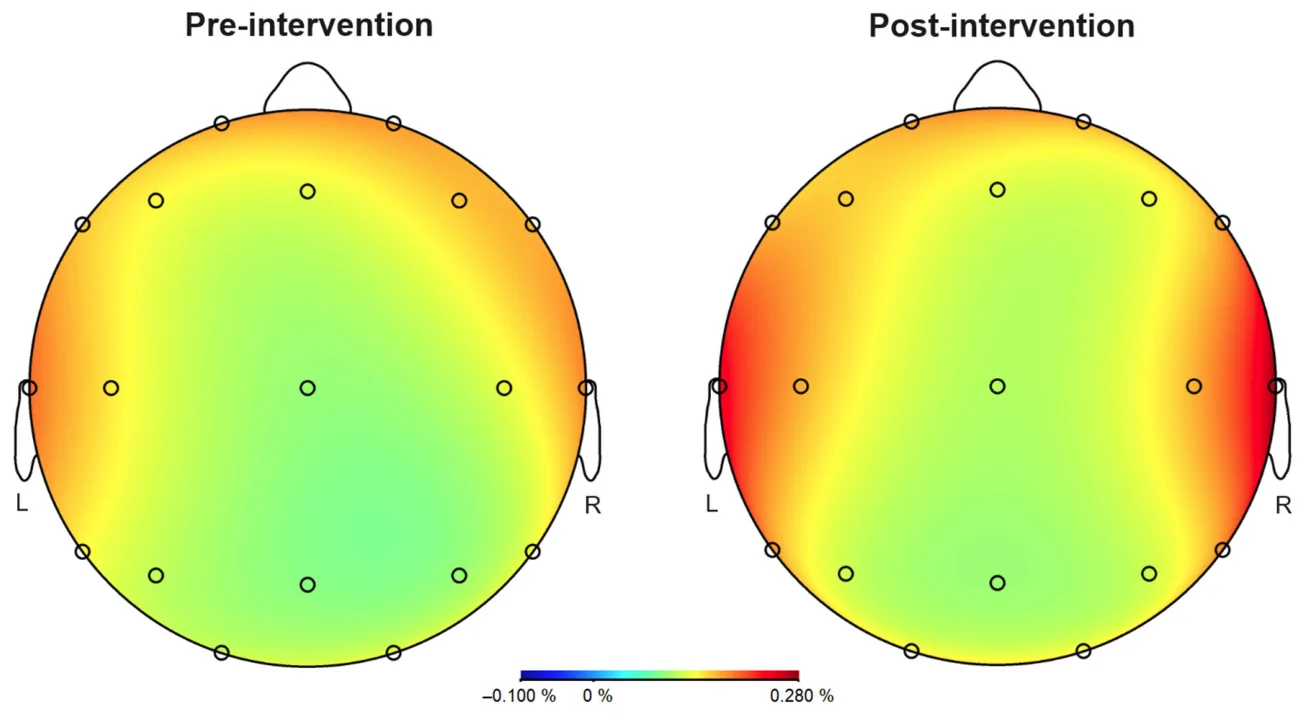
Topographic distribution of resting-state 38–42 Hz relative power before and after the intervention. Scalp topographic maps show the group-averaged distribution of resting-state relative spectral power in the pre- and post-intervention conditions. Relative power was quantified as the mean relative spectral power across the predefined 38–42 Hz frequency range. L = left; R = right.

**Figure 3:**
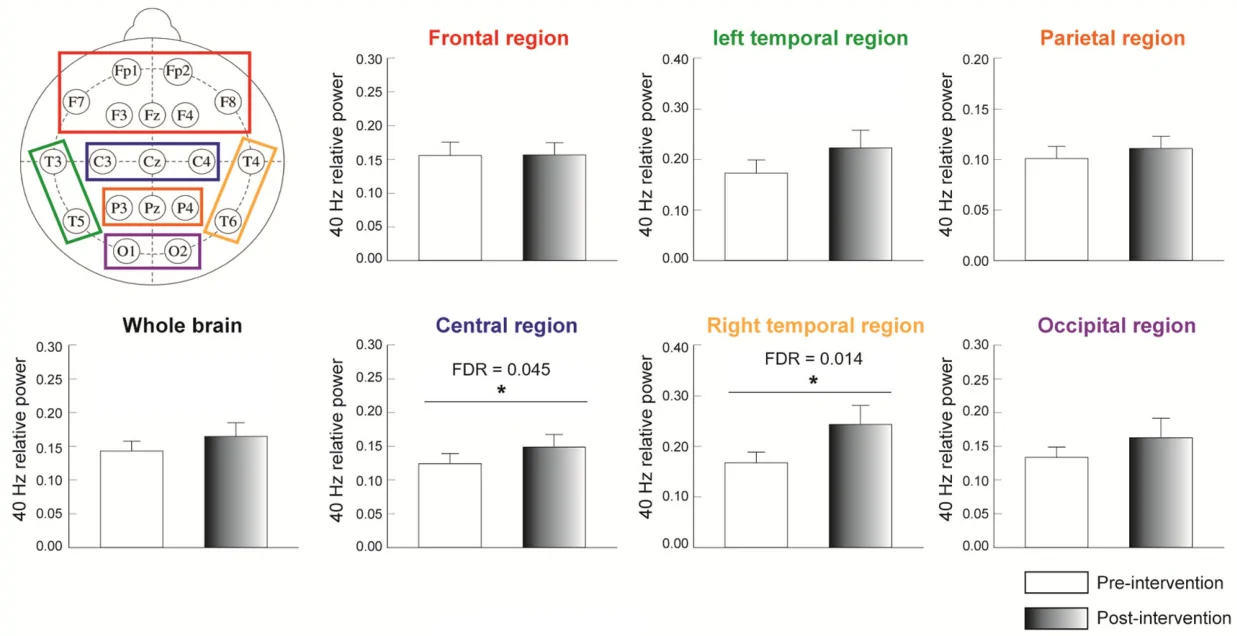
Pre- and post-intervention resting-state 38–42 Hz relative power across scalp regions. Scalp electrodes were grouped into six predefined regions: frontal (red), central (blue), parietal (orange), left temporal (green), right temporal (yellow), and occipital (purple). Resting-state 38–42 Hz relative power was defined as the mean relative spectral power across the predefined 38–42 Hz frequency range. Following correction for multiple comparisons using the Benjamini–Hochberg false discovery rate (FDR) procedure, significant increases in relative power were observed in the central and right temporal regions following the intervention. Error bars represent the standard error of the mean (SEM). * FDR < 0.05.

Figure 2 illustrates the topographic distributions of 38–42 Hz relative power averaged across all participants before and after the intervention. Following correction for multiple comparisons, significant increases in 38–42 Hz relative power were observed in the central region (*Z* = 2.223, *p* = 0.013, FDR = 0.045, effect size = 0.555; HL estimate = 0.023, 95% CI [0.003, 0.045]) and the right temporal region (*Z* = 2.896, *p* = 0.002, FDR = 0.014, effect size = 0.724; HL estimate = 0.063, 95% CI [0.016, 0.118]) following the 60-min intervention (Figure 3, Table 2). Nominal increases were also observed in the left temporal (*p* = 0.024) and whole-brain (*p* = 0.039) measures; however, these effects did not remain statistically significant after FDR correction. To provide broader spectral context for these findings, resting-state EEG relative power spectra across the 30–50 Hz frequency range are presented for the pre- and post-intervention conditions in Supplementary Figure S1.

As a sensitivity analysis, absolute 38–42 Hz power was examined to assess the robustness of the relative-power findings to the normalization procedure. Following correction for multiple comparisons, a significant increase in absolute 38–42 Hz power was observed in the right temporal scalp region (*Z* = 2.482, *p* = 0.006, FDR = 0.042, effect size = 0.620; HL estimate = 0.049, 95% CI [0.011, 0.108]) following the 60 min intervention. Nominal increases were also observed in the central (*p* = 0.024), parietal (*p* = 0.039), and left temporal (*p* = 0.028) regions; however, these differences did not remain statistically significant after FDR correction. Complete results of the absolute-power sensitivity analysis are provided in Supplementary Table S1.

Of the 16 participants, only one (6.3%) reported experiencing very mild fatigue after a single 60 min session of 40 Hz non-flickering light stimulation.

**Figure 4:**
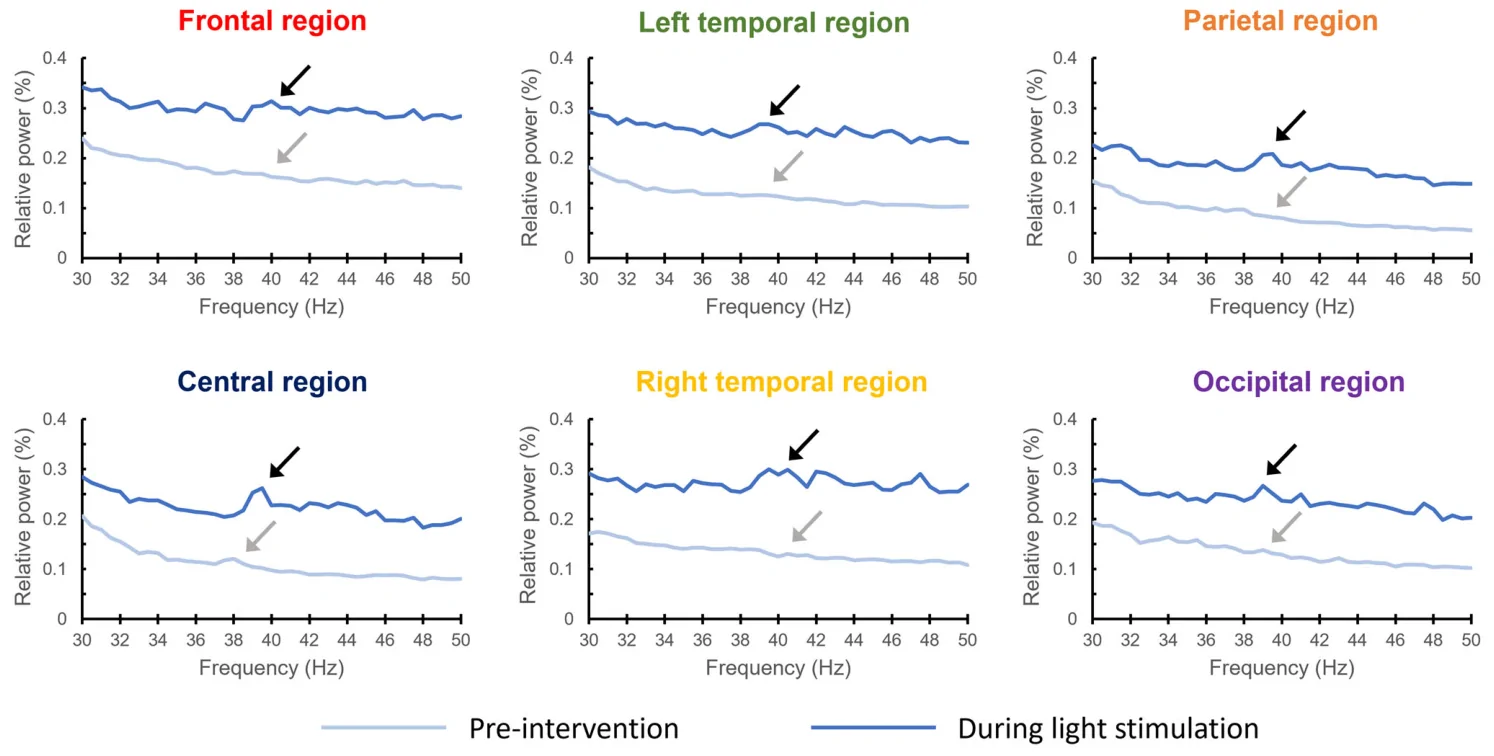
P Power spectral density before and during non-flickering 40 Hz light stimulation in the real-time EEG subset (n = 10). Power spectral density (PSD) is shown for the pre-stimulation (light blue) and during-stimulation (dark blue) conditions across the predefined scalp regions and whole-brain measure. Black arrows indicate visually apparent spectral prominence within the predefined 38–42 Hz target range during stimulation, whereas gray arrows indicate the corresponding frequency range in the pre-stimulation condition. The spectra are presented as a qualitative visualization of spectral activity around the target stimulation frequency. Quantitative comparisons of 38–42 Hz signal-to-noise ratio (SNR) between the pre-stimulation and during-stimulation conditions are provided in Supplementary Table S2.

Real-time EEG recordings obtained during 40 Hz stimulation were available for a subset of 10 participants. Visual inspection of the power spectra showed spectral prominence around the target 38–42 Hz range during stimulation in several scalp regions (Figure 4). To quantitatively assess this observation without relying on visually selected spectral peaks, SNR within the predefined 38–42 Hz range was compared between the pre-stimulation and during-stimulation conditions. SNR values were descriptively higher during stimulation across all six scalp regions and the whole-brain measure. The increase reached nominal significance in the right temporal region (pre-stimulation: 0.978 ± 0.016; during stimulation: 1.087 ± 0.044; *Z* = 1.988, one-tailed *p* = 0.026), whereas the remaining regional and whole-brain comparisons did not reach nominal significance (all *p* > 0.05; Supplementary Table S2). These exploratory findings indicate increased spectral prominence around the target frequency during stimulation in some scalp regions; however, they do not establish generalized neural entrainment or distinguish neural activity from potential stimulation-related artifact.

## 4. Discussion

The present uncontrolled single-arm pilot study examined changes in resting-state 38–42 Hz EEG activity following a single 60 min session of non-flickering 40 Hz light stimulation in cognitively normal older adults. After correction for multiple comparisons, significant increases in resting-state 38–42 Hz relative power were observed over the central and right temporal scalp regions. In the absolute-power sensitivity analysis, the right temporal increase remained significant after FDR correction, providing stronger support for the robustness of the right temporal finding while warranting greater caution in interpreting the central finding. Separately, in the exploratory subset, SNR values during stimulation were descriptively higher than pre-stimulation values across all regions, with only the right temporal comparison reaching nominal significance (*p* = 0.026). This exploratory during-stimulation observation should be considered distinct from the post-stimulation resting-state finding and does not provide independent confirmation of it. The intervention was well tolerated, with only one participant reporting very mild fatigue. Taken together, these findings provide preliminary electrophysiological evidence of altered resting-state 38–42 Hz activity following non-flickering 40 Hz light stimulation, while the uncontrolled design and small sample preclude conclusions regarding causality or regional specificity.

Previous studies have employed a wide range of outcome measures to evaluate the efficacy of 40 Hz sensory stimulation in older adults or individuals with cognitive decline, including gray matter volume, white matter integrity, functional connectivity from functional magnetic resonance imaging (fMRI), cognitive performance, and neuropsychiatric symptoms [11,12,13,14,15,17]. However, these studies have largely focused on outcomes other than resting-state electrophysiological changes following stimulation. The present study provides preliminary EEG evidence of changes in resting-state 40 Hz spectral activity following a single session of non-flickering 40 Hz light stimulation in cognitively normal older adults. Whether such electrophysiological changes contribute to the structural, functional, or cognitive benefits reported in previous studies remains unknown. One possibility is that modulation of gamma oscillatory activity may represent a neurophysiological mechanism contributing to some of these effects; however, this interpretation remains speculative and cannot be evaluated by the present study. Future controlled studies integrating EEG with other outcome measures (e.g., neuropsychological tests, sleep metrics, neuroimaging, and biomarkers) are needed to determine whether changes in gamma activity are associated with, or potentially contribute to, longer-term functional and clinical outcomes.

A potential concern is whether a single 60 min session is sufficient to induce measurable neural changes in humans. Evidence from related paradigms supports this possibility. For example, one study with 20 healthy young adults showed that just 10 min of 40 Hz auditory stimulation significantly increased corticospinal excitability, as measured by motor evoked potentials following transcranial magnetic stimulation over the primary motor cortex [21]. Another fMRI study reported that only 5 min of 40 Hz transcranial vibration stimulation enhanced spontaneous brain activity, as indexed by increased amplitude of low-frequency fluctuations (ALFF) post-intervention [20]. Together with our findings, these findings indicate that measurable changes in brain activity can be observed following relatively brief periods of 40 Hz sensory stimulation, although the mechanisms and specificity of these acute changes remain to be established.

In the present study, significant increases in resting-state 38–42 Hz relative power were observed over the central and right temporal scalp regions after correction for multiple comparisons, whereas no significant change was detected over the occipital region. To assess whether these findings might reflect changes in the total spectral power used for normalization, we additionally examined absolute 38–42 Hz power. The right temporal increase remained significant after FDR correction, suggesting that this finding cannot be explained solely by changes in total 1–100 Hz power. In contrast, the absolute-power increase over the central region did not remain significant after correction and should therefore be interpreted more cautiously. Importantly, given the small sample size, nonsignificant findings in the occipital and other regions may reflect limited statistical power and should not be considered evidence of regional specificity. Moreover, scalp-recorded EEG, particularly with a relatively low-density montage, cannot identify the underlying cortical generators. Thus, the central and right temporal findings should be interpreted as scalp-level changes rather than preferential engagement of the corresponding cortical areas, and the absence of an occipital effect does not indicate a lack of visual-cortex involvement. Future studies with larger samples and source-resolved high-density EEG, MEG, or complementary neuroimaging are needed to determine the reproducibility and neural sources of these spatial patterns.

The eyes-closed resting-state condition should also be considered when interpreting the present findings. This condition was selected to minimize ongoing visual inputs and eye-movement-related variability and was applied consistently before and after stimulation. Nevertheless, eyes-closed resting states are characterized by prominent alpha activity [26,27,28], which may influence the overall spectral composition and consequently relative power estimates across frequency bands. Although the 38–42 Hz range is well separated from the conventional alpha band, potential cross-frequency interactions and changes in spectral composition cannot be excluded [29,30]. Accordingly, the observed post-stimulation increases in 38–42 Hz relative power should be interpreted as changes in eyes-closed resting-state spectral activity rather than as evidence of persistent 40 Hz visual entrainment. Future studies incorporating both eyes-open and eyes-closed recordings may help determine whether these post-stimulation changes depend on resting-state conditions.

Several limitations should be acknowledged. First and most importantly, the present study employed an uncontrolled single-arm pre–post design. Therefore, the observed changes cannot be attributed specifically to the 40 Hz stimulation, as potential contributions from nonspecific light exposure, variations in conversational engagement, gaze direction, and effective visual exposure during the intervention, the passage of time, changes in alertness or arousal, repeated EEG assessment, and other sources of recording-related variability cannot be excluded. The ongoing randomized controlled trial includes an active control condition receiving non-flickering light stimulation at an alternative frequency, which will permit direct between-group comparisons and help distinguish changes associated with 40 Hz stimulation from nonspecific intervention effects and spontaneous temporal fluctuations in resting-state EEG. The complete results of the registered trial will be reported upon study completion. Second, the single-session design and relatively small sample size limit the generalizability of the findings and the statistical power to detect smaller pre–post differences. Consequently, nonsignificant findings in individual scalp regions should not be interpreted as evidence of the absence of neurophysiological change or as establishing regional specificity. The present study also does not allow conclusions regarding long-term efficacy or clinical significance. Third, although increases in 40 Hz power were observed in the central and right temporal regions after correction for multiple comparisons, source-level analyses were not performed; thus, the precise neural generators underlying these scalp-level changes remain uncertain. In addition, dedicated electromyographic (EMG) recordings were not obtained. Although ICA-based artifact removal was applied during EEG preprocessing to identify and remove components exhibiting characteristics of muscle activity, residual myogenic contamination cannot be completely excluded, particularly at temporal electrodes located near the temporalis muscles. It should also be noted that because EEG was recorded concurrently with light stimulation in the subset of participants included in the exploratory real-time EEG assessment, the observed 38–42 Hz spectral prominence during stimulation cannot definitively be attributed to neural entrainment or distinguished from potential stimulation-related artifacts. Accordingly, the observed right temporal 38–42 Hz increase should be interpreted cautiously. Fourth, although electrode impedance was maintained below 1 MΩ before data acquisition according to the procedures used for the DSI-24 dry-electrode system, impedance was not continuously monitored throughout the recordings. Therefore, potential recording variability related to electrode–scalp contact or impedance changes cannot be completely excluded, which is particularly relevant when interpreting activity in the 38–42 Hz frequency range. Finally, the study focused solely on resting-state EEG and did not assess pre–post behavioral or cognitive outcomes, which limits our ability to determine whether the observed electrophysiological changes were associated with functional benefits.

## 5. Conclusions

In conclusion, this pilot EEG study provides preliminary evidence of regional increases in resting-state 38–42 Hz activity following a single session of non-flickering 40 Hz light stimulation in cognitively normal older adults, with the right temporal finding showing additional support from the absolute-power sensitivity analysis. The stimulation was well tolerated, supporting the feasibility of this non-flickering approach. However, because of the absence of a concurrent control condition, the observed pre–post changes cannot be attributed specifically to 40 Hz stimulation. These findings provide a basis for controlled studies examining the reproducibility, frequency specificity, and functional relevance of the observed electrophysiological changes.

## Figures and Tables

**Table 1:** Demographic information and neuropsychological performance of the included subjects (n = 16).

Item	Mean ± SEM	Median [Q1, Q3]
Age (ages)	70.56 ± 2.18	70 [65, 76]
Education (years)	11.43 ± 0.92	12 [9, 16]
CASI	93.06 ± 1.54	95 [92, 98]
GDS	0.31 ± 0.17	0 [0, 0]
CVVLT		
Total score	26.25 ± 1.06	26 [24, 30]
Immediate recall	7.62 ± 2.71	8 [7, 9]
Delayed recall	7.50 ± 2.58	8 [7, 8]
Logic Memory		
Immediate recall	13.81 ± 1.39	15 [13, 18]
Delayed recall	12.62 ± 1.40	14 [10, 17]
Complex Figure Test		
Copy	32.84 ± 1.29	34 [34, 36]
Immediate recall	21.23 ± 1.76	19 [15, 28]
Delayed recall	20.76 ± 1.54	19.5 [14, 26.5]
Digit Span Test		
Forward	7.75 ± 0.39	9 [6, 9]
Backward	4.93 ± 0.34	5 [4, 7]
Verbal Fluency Test (correct trials)	18.37 ± 1.16	19 [16, 23]
Trail Making Test–A (seconds)	66.18 ± 11.99	50 [38, 66]
Trail Making Test–B (seconds)	94.68 ± 12.65	79 [61, 107]

SEM = standard error of the mean; Q1 = 25th percentile; Q3 = 75th percentile; CASI = Cognitive Abilities Screening Instrument; GDS = Geriatric Depression Scale; CVVLT = Chinese Version Verbal Learning Test.

**Table 2:** Pre–post comparisons of resting-state 38–42 Hz relative power across scalp regions.

Region	Pre-Intervention, Mean ± SEM	Pre-Intervention, Median [Q1, Q3]	Post-Intervention, Mean ± SEM	Post-Intervention, Median [Q1, Q3]	*Z*	Effect Size	*p*	FDR	HL Estimate [95% CI]
Whole brain	0.143 ± 0.015	0.147 [0.095, 0.203]	0.165 ± 0.020	0.149 [0.097, 0.227]	1.758	0.439	0.039	0.068	0.019 [−0.003, 0.041]
Frontal	0.156 ± 0.020	0.140 [0.095, 0.229]	0.157 ± 0.018	0.164 [0.093, 0.214]	0.465	0.116	0.321	0.321	0.003 [−0.024, 0.027]
Central	0.124 ± 0.015	0.120 [0.077, 0.156]	0.149 ± 0.019	0.135 [0.082, 0.194]	2.223	0.555	0.013	0.045 *	0.023 [0.003, 0.045]
Parietal	0.101 ± 0.012	0.095 [0.060, 0.118]	0.111 ± 0.012	0.096 [0.077, 0.150]	1.138	0.284	0.127	0.177	0.011 [−0.005, 0.029]
Left temporal	0.173 ± 0.026	0.165 [0.091, 0.224]	0.223 ± 0.035	0.191 [0.100, 0.377]	1.965	0.491	0.024	0.056	0.039 [−0.001, 0.095]
Right temporal	0.168 ± 0.021	0.161 [0.089, 0.218]	0.244 ± 0.037	0.218 [0.112, 0.373]	2.896	0.724	0.002	0.014 *	0.063 [0.016, 0.118]
Occipital	0.134 ± 0.015	0.121 [0.094, 0.179]	0.163 ± 0.029	0.109 [0.072, 0.229]	0.827	0.206	0.204	0.238	0.011 [−0.014, 0.084]

Values are presented as mean ± standard error of the mean (SEM) and median [Q1–Q3], where Q1 and Q3 represent the 25th and 75th percentiles, respectively. Pre–post comparisons were performed using one-tailed Wilcoxon signed-rank tests based on the a priori directional hypothesis of increased resting-state 38–42 Hz relative power following stimulation. *p* values were adjusted across the seven EEG measures using the Benjamini–Hochberg false discovery rate (FDR) procedure. Effect sizes were calculated as *r* = *Z*/√*N*. HL estimates represent Hodges–Lehmann estimates of the paired pre–post difference (post-intervention minus pre-intervention), with corresponding 95% confidence intervals (CI). Positive HL estimates indicate higher values at post-intervention. * FDR < 0.05.

## Data Availability

The data presented in this study are available upon request from the corresponding author. The data are not publicly available due to ethical restrictions. Additional details regarding the trial protocol and planned statistical analyses are also available from the corresponding author.

## Supplementary Materials

The following supporting information can be downloaded at: https://www.mdpi.com/article/10.3390/brainsci16090976/s1, Figure S1: Resting-state EEG relative power spectra from 30 to 50 Hz before and after the intervention; Table S1: Sensitivity analysis of pre–post changes in resting-state 38–42 Hz absolute power across scalp regions; Table S2: Exploratory comparison of 38–42 Hz signal-to-noise ratio between pre-stimulation and during-stimulation conditions.
