# Supplementary material for: Resting-State 40 Hz EEG Activity Before and After Single-Session Non-Flickering 40 Hz Light Stimulation in Cognitively Normal Older Adults: An Uncontrolled Pilot Study
Source: Brain Sci. 2026 Sep 15;16(9):976. doi: 10.3390/brainsci16090976 (PMC13604170; doi:10.3390/brainsci16090976)
Supplement: Supplementary file 1 [file brainsci-16-00976-s001.zip › Supplementary Figure S1.pdf]

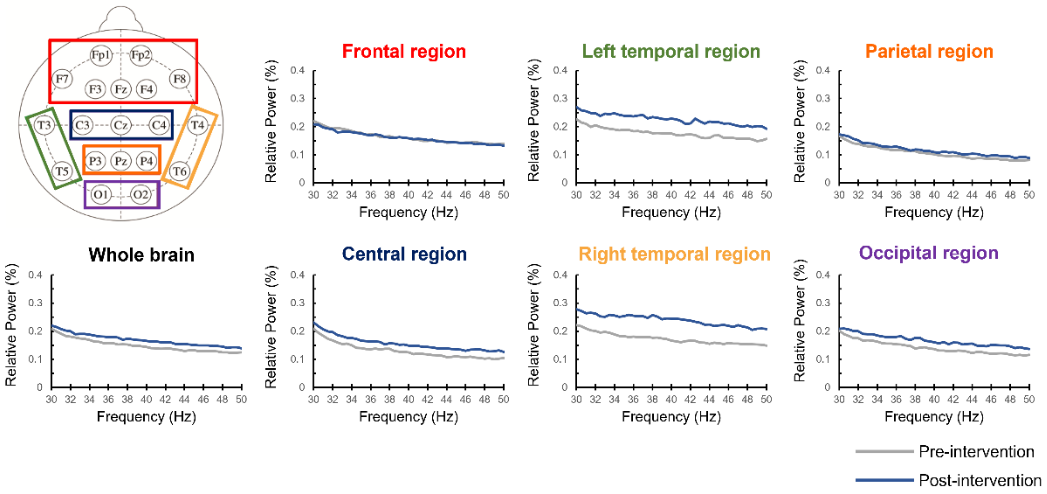

Supplementary Figure S1. Resting-state EEG relative power spectra from 30 to 50 Hz before and after the intervention. Group-averaged relative power spectra are shown for the pre-intervention (gray) and post-intervention (blue) conditions across the six predefined scalp regions (frontal, central, parietal, left temporal, right temporal, and occipital) and the whole-brain measure. Spectra are displayed over the 30–50 Hz frequency range to illustrate the spectral distribution surrounding the predefined 38–42 Hz target range.
