# Supplementary material for: Resting-State 40 Hz EEG Activity Before and After Single-Session Non-Flickering 40 Hz Light Stimulation in Cognitively Normal Older Adults: An Uncontrolled Pilot Study
Source: Brain Sci. 2026 Sep 15;16(9):976. doi: 10.3390/brainsci16090976 (PMC13604170; doi:10.3390/brainsci16090976)
Supplement: Supplementary file 1 [file brainsci-16-00976-s001.zip › Supplementary Table S1.pdf]

Supplementary Table S1. Sensitivity analysis of pre–post changes in resting-state 38–42 Hz absolute power across scalp regions

**Region**	**Pre-intervention, mean ± SEM**	**Pre-intervention, median [Q1, Q3]**	**Post-intervention, mean ± SEM**	**Post-intervention, median [Q1, Q3]**	***Z***	**Effect size**	***p***	**FDR**	**HL estimate [95% CI]**
Whole brain	0.082 ± 0.011	0.063 [0.043, 0.124]	0.095 ± 0.012	0.099 [0.043, 0.148]	1.241	0.310	0.107	0.149	0.009 [−0.004, 0.031]
Frontal	0.107 ± 0.020	0.075 [0.045, 0.129]	0.093 ± 0.015	0.069 [0.048, 0.143]	0.776	0.194	0.219	0.219	−0.010 [−0.049, 0.018]
Central	0.071 ± 0.011	0.056 [0.032, 0.114]	0.086 ± 0.015	0.067 [0.042, 0.112]	1.965	0.491	0.024	0.065	0.012 [−0.0001, 0.034]
Parietal	0.056 ± 0.010	0.046 [0.030, 0.064]	0.059 ± 0.006	0.056 [0.036, 0.068]	1.758	0.439	0.039	0.068	0.006 [−0.001, 0.014]
Left temporal	0.085 ± 0.019	0.056 [0.028, 0.114]	0.131 ± 0.030	0.077 [0.030, 0.242]	1.913	0.478	0.028	0.065	0.035 [−0.0002, 0.074]
Right temporal	0.069 ± 0.013	0.048 [0.030, 0.100]	0.135 ± 0.028	0.110 [0.033, 0.180]	2.482	0.620	0.006	**0.042***	0.049 [0.011, 0.108]
Occipital	0.064 ± 0.008	0.053 [0.036, 0.089]	0.092 ± 0.018	0.066 [0.049, 0.097]	1.086	0.271	0.139	0.162	0.009 [−0.010, 0.062]

Values are presented as mean ± standard error of the mean (SEM) and median [Q1–Q3], where Q1 and Q3 represent the 25th and 75th percentiles, respectively. Pre–post comparisons were performed using one-tailed Wilcoxon signed-rank tests based on the a priori directional hypothesis of increased resting-state 38–42 Hz absolute power following stimulation. The *p* values were adjusted across the seven EEG measures using the Benjamini–Hochberg false discovery rate (FDR) procedure. Effect sizes were calculated as $r = Z / \sqrt{N}$. HL estimates represent Hodges–Lehmann estimates of the paired pre–post difference (post-intervention minus pre-intervention), with corresponding 95% confidence intervals (CI). Positive HL estimates indicate higher values at post-intervention.

*FDR < 0.05
