# Supplementary material for: Resting-State 40 Hz EEG Activity Before and After Single-Session Non-Flickering 40 Hz Light Stimulation in Cognitively Normal Older Adults: An Uncontrolled Pilot Study
Source: Brain Sci. 2026 Sep 15;16(9):976. doi: 10.3390/brainsci16090976 (PMC13604170; doi:10.3390/brainsci16090976)
Supplement: Supplementary file 1 [file brainsci-16-00976-s001.zip › Supplementary Table S2.pdf]

Supplementary Table S2. Exploratory comparison of 38–42 Hz signal-to-noise ratio between pre-stimulation and during-stimulation conditions

**Region**	**Pre-stimulation, mean ± SEM**	**Pre-stimulation, median [Q1, Q3]**	**During-stimulation, mean ± SEM**	**During-stimulation, median [Q1, Q3]**	***Z***	***p***
Whole brain	0.975 ± 0.012	0.974 [0.950, 1.009]	1.042 ± 0.036	1.030 [0.973, 1.070]	1.580	0.057
Frontal	0.980 ± 0.014	0.973 [0.938, 1.019]	1.022 ± 0.032	1.019 [0.952, 1.071]	0.663	0.254
Central	0.956 ± 0.019	0.971 [0.909, 0.994]	1.064 ± 0.067	1.004 [0.949, 1.083]	1.376	0.084
Parietal	0.964 ± 0.023	0.967 [0.915, 1.033]	1.075 ± 0.060	1.022 [0.946, 1.203]	1.274	0.101
Left temporal	0.987 ± 0.014	0.984 [0.953, 1.020]	1.015 ± 0.023	1.035 [0.927, 1.068]	1.376	0.084
Right temporal	0.978 ± 0.016	0.958 [0.941, 1.013]	1.087 ± 0.044	1.033 [0.997, 1.174]	1.988	0.026
Occipital	0.998 ± 0.013	1.000 [0.967, 1.024]	1.052 ± 0.039	1.052 [0.941, 1.147]	1.274	0.101

Values are presented as mean ± standard error of the mean (SEM) and median [Q1–Q3], where Q1 and Q3 represent the 25th and 75th percentiles, respectively. Pre-stimulation and during-stimulation SNR values were compared using one-tailed Wilcoxon signed-rank tests based on the a priori directional hypothesis of higher 38–42 Hz spectral prominence during stimulation. Because these analyses were conducted as an exploratory technical assessment in a subset of participants (n = 10), unadjusted p values are reported.
